# Surveillance of Social and Geographic Inequalities in Housing-Related Issues: The Case of the Eastern Townships, Quebec (Canada) 

**DOI:** 10.3390/ijerph110504825

**Published:** 2014-05-05

**Authors:** Mathieu Roy, Mélissa Généreux, Émélie Laverdière, Alain Vanasse

**Affiliations:** 1 Faculty of Medicine and Health Sciences, Université de Sherbrooke, Sherbrooke, Québec J1H 5N4, Canada; E-Mails: emelie.laverdiere@usherbrooke.ca (E.L.); alain.vanasse@usherbrooke.ca (A.V.); 2 Eastern Townships Public Health Department, Université de Sherbrooke, Sherbrooke, Québec J1G 1B1, Canada, Canada; E-Mail: mgenereux.agence05@ssss.gouv.qc.ca

**Keywords:** environmental health, epidemiological surveillance, social conditions, socioeconomic factors, geography, medical, inequalities, public health

## Abstract

Even though health inequalities are conditioned by many aspects of the environment, much of the existing research focuses on the social environment. This emphasis has the effect to neglect other environmental aspects such as its physical dimension. The physical environment, which is linked to housing conditions, may contribute to the uneven distribution of health. In this study, we examined 19 housing-related issues among a representative sample of 2,000 adults residing in a Quebec (Canada) health region characterized by a mix of rural, semi-rural, and urban areas. The distribution of these issues was examined according to socioeconomic and geographic indicators of social position. Summary measures of inequalities were assessed. Our results showed that the prevalence of nearly all housing-related issues was higher among low-income households compared to more affluent ones. Highly educated individuals showed better housing conditions, whereas different issues tended to cluster in deprived or densely populated areas. To conclude, we observed steep gradients between social class and poor housing conditions. This may explain a substantial part of health inequality on the regional scale. The surveillance of housing-related issues is therefore essential to properly inform and mobilize local stakeholders and to develop interventions that target vulnerable groups on this level.

## 1. Introduction

The field of health promotion has significantly contributed to the analysis of disparities in individual behavior and in access to health care. However, it has been well established that inequalities in health cannot be fully understood with these determinants alone [1,2]. Environment is also a major factor in the production of health inequalities [2]. While many aspects of the environment have been suggested to play a role in the uneven distribution of health [2,3], most of the existing research focuses on aspects related to social environment (e.g., social support, social capital, criminality, perceived security). This scientific emphasis must however also consider other environmental features, such as those related to physical aspects which are also a cornerstone for health and its unequal distribution within populations [4,5,6,7].

### 1.1. Physical Environment, Housing and Residential Conditions, and Health Inequalities

Physical environment is intrinsically linked with housing and residential conditions [3,8], which in turn affect health and its distribution across populations [4,5,6,7,9,10]. This pathway linking physical environment and disparities in health is not surprising when we learn that the average individual spends two third of their life in their home environment [11]. This proportion can even reach 80% among most vulnerable groups such as children, seniors, and unemployed adults [12]. The environment where people spend most of their time influences the air they breathe, the water and the food they consume, the sounds they hear, the stress they experience, and many other life aspects. All of these exposure types are largely determined by the housing and residential conditions [3,7,13]. 

Poor housing conditions have been associated with increased likelihood of accidents and injuries [14], higher mortality [15,16], higher rates of respiratory symptoms [17,18], cardiovascular complications [19], and mental health problems [20,21]. Not only do poor housing conditions increase the incidence of adverse health outcomes, it can also exacerbate pre-existing medical conditions. For instance, the presence of physical (e.g., humidity), chemical (e.g., wood and tobacco smoke), and biological agents (e.g., allergens, molds) in indoor air may trigger symptoms of asthma or rhinitis [17,18]. Many other environmental hazards related to residential conditions (*i.e.*, neighborhood characteristics), such as industrial or traffic-related air and/or noise pollution, location in an urban heat island [7,22]), or structural defects in dwelling units (e.g., poor water quality, insulation [23]) may also be responsible of poorer health outcomes.

In addition to evidence linking housing and residential conditions to health, there is a strong and positive correlation between these conditions, and dwelling unit prices [4,7,24]. This contributes to social and geographic inequalities in housing or residential-related health outcomes [7,10]. More economically-deprived households tend to cluster in neighborhoods where housing prices are cheaper. People living in low-income areas are more exposed to more detrimental housing conditions and physical environments [4]. This phenomenon has been called “*residential segregation*” and it was observed in Canada [25] and in United States [26]. It adds to the already precarious living conditions of more vulnerable groups. Aside from being exposed to higher levels of housing-related health hazards, poorly educated and less fortunate people may also be affected to a greater extent when exposed to these types of hazards, and be less able to cope with the associated health consequences, two factors that contribute to their vulnerability in its broadest sense. 

In sum, different associations were observed between social position, and: (1) housing conditions, (2) residential conditions, and (3) health outcomes related to these conditions (hereafter named housing-related issues [4]). Social position has also been found to exacerbate pre-existing housing-related issues in two different ways: (1) exposures may vary according to social position, and (2) given certain level of exposures, social position may modify the effects on health of such exposure [27].

### 1.2. The World Health Organization (WHO) Health Inequalities Surveillance Framework

In 2008, the WHO *Commission on the Social Determinants of Health* raised the need to better assess social inequalities in health through a surveillance system that relies on the selection of appropriate: (1) social indicators, (2) health (or health determinants) indicators, and (3) summary measures of inequalities [28]. This framework applied to housing-related issues may serve to plan and evaluate interventions which tackle health inequalities.

With respect to social indicators, measures that capture the complementary aspects of social position should be used instead [29]. Indicators such as socioeconomic status (*e.g.*, education, income, occupation) and geographic location (e.g., place of residence, neighborhood characteristics) also have to be considered [28]. In the scientific literature, income is correlated to social position which is commonly associated with housing-related issues [30]. As previously mentioned, better housing conditions are strongly linked to higher dwelling prices [4,7,24]. With respect to health (or health determinants) indicators, the greatest majority of evidence concerning housing and residential conditions as predictors of adverse health outcomes includes heating systems, extreme temperatures, and environmental tobacco smoke [3]. Emerging issues such as community or traffic-related noise are also increasingly recognized as factors affecting health [4,31,32]. With respect to the last aspect of the WHO’s framework, most studies have described the extent of health inequalities across social classes using simple summary measures such as prevalence ratios and differences [24,33,34,35,36,37,38]. More complex measures have also been used [29] but these may be less relevant to inform and mobilize stakeholders that need clear and easily understandable information.

### 1.3. The Current Study

Although some authors have already studied associations between social position and specific features of the physical environment including housing-related issues [39,40,41,42,43], very few have thoroughly examined their uneven distribution within populations [7]. The rare studies that have done so were from European countries. Other industrialized countries such as Canada lag behind. These studies further used national or supra-national data despite growing evidence that wide inequalities also exist on smaller scales [7,31]. The uneven distribution of housing-related issues as a function of social position on a regional level remains a major public health challenge that needs to be better understood. Because health disparities exist between countries, but also between and within communities [30], public health efforts to monitor the magnitude of these inequalities should be undertaken in smaller settings such as regional and local ones. This is important to gather relevant information aiming to develop public health strategies to reduce such inequalities and to raise awareness among local decision-makers about specific needs of their population. Regional surveillance of housing-related issues should be particularly helpful to influence municipalities and other local partners who can provide housing opportunities that meet the needs of all citizens, especially the most disadvantaged ones.

In an effort to fill these gaps in the scientific literature, the aim of this study was to apply the WHO health inequalities surveillance framework on regional and local scales to efficiently support public health actions and policies to tackle inequalities related to various housing and residential conditions. To meet this goal, our main research objective was to examine the social gradient of a wide range of housing-related issues, using various indicators of social position. We expect that strong gradients will be observed on regional and local scales for most of the housing-related issues examined.

## 2. Methods

### 2.1. Setting

In the province of Quebec (Canada), health and social services are regrouped into one single administration (*i.e.*, the Quebec Health and Social Services Ministry). This central administration is divided along 18 health regions (or health authorities) which in turn are divided along 95 health and social services territories. This governance model allows to local stakeholders to become responsible of their population and to administer and/or deliver health care to their residents.

This study was conducted in one of these 18 Quebec (Canada) health regions known as the *Eastern Townships* which are a mix of urban, semi-urban, and rural communities with a population around 315,000 individuals. Among this population, about half live in one central city named Sherbrooke (Quebec’s 6th largest city [44]). The Eastern Townships health region was divided into 66 local communities (33 in Sherbrooke and 33 outside this city) by the regional public health authority in partnership with local stakeholders. These local communities were defined in 2008 using a participatory approach involving sixty actors in community development. These local communities are smaller than health and social services territories. They were defined with the specific intent to create relatively homogeneous territories with respect to socioeconomic characteristics of their residents. Each community has about 5,000 residents.

The Eastern Townships are appropriate to explore inequalities related to housing and residential conditions. This region is indeed a mix of geographical and social characteristics. It includes one central university town surrounded by semi-rural and rural areas. A wide range of socioeconomic situations is also observed, including: (1) young academics, (2) relatively affluent professionals mainly working for health and education sectors, and (3) less affluent or educated individuals from the downtown areas or rural farms. In short, this region is a microcosm in which findings may be potentially generalizable to other Canadian regions and elsewhere in Western industrialized settings.

### 2.2. Sample and Recruitment Procedures

In September 2011, a cross-sectional random digit-dial telephone survey was conducted among a representative sample of 2,000 adults (≥18 years old) living in Eastern Townships in private households. This sample was stratified according to the city of residence with half of participants living in Sherbrooke and the remainder living elsewhere in this health region. 

The sampling frame used for recruitment was a residential phone number database. Numbers corresponding to mobile phones were excluded. Recruitment of participants was performed according to three steps. First, private households were randomly selected. They were then selected as a function of (1) being located in Eastern Townships and (2) being composed of at least one adult. In the third step of recruitment process, one adult was randomly selected from each eligible household. Selected adults could not be substituted. If they were unavailable at this stage of the process, interviewers called them back at the most convenient time for them. Participants were asked to answer a short questionnaire. Data collection was conducted by an independent and experienced firm of research and survey. All interviewers were trained and aware of standardization procedure for telephone survey administration. The response rate was 63.5%, which is high for random digit-dial telephone surveys [45], and sufficient to reduce potential biases related to non-response rates [46,47].

### 2.3. The Questionnaire

The questionnaire used in this study was available in both French and English and contained questions assessing exposure to various housing-related issues. It also captured participants’ social position with different socioeconomic and geographic indicators. The questionnaire was revised and translated by computerized polling firm. Translations from French to English were reviewed by an external translator using reverse translation method. Both versions of the questionnaire were pre-tested with 20 individuals. This step helped to validate understanding of the issues examined and allowed us to add response categories that were not initially foreseen.

### 2.4. Measures

#### 2.4.1. Housing-related Issues

A total of 19 housing-related issues were assessed (Table 1). Such issues were measured using the questionnaire with the exception of: (1) being located in an urban heat island, and (2) living in an Eastern Township local community with high density of high-traffic roadways. For these two outcomes, we coupled the six-digit postal code confirmed by participants during their interview to the 2011 Canadian census data. Housing-related issues were grouped according to five dimensions of physical environment: (1) carbon monoxide (CM), (2) wood burning, (3) heat stress, (4) environmental tobacco smoke (ETS), and (5) community noise. Questions used to assess housing-related issues were described in Table 1 and have already been used in previous national or regional surveys [48,49,50,51,52].

**Table 1:** Examined housing-related issues (*n* = 19) regrouped along five dimensions of physical environment.

Housing-related Issues	Question (Answers)
**Dimension 1: CM**	
1. CM source (s)	Presence of ≥ one source of CM at-home, including indoor garage, stove or heating systems using fuel (yes, no).
2. Lack of knowledge of CM source (s)	Adequate knowledge of CM sources (yes, no).
3. No CM detector	Absence of CM detector in participant residence (yes, no).
4. No CM detector among houses with CM source (s)	Absence of CM detector residence of individuals with a source of CM at-home (yes, no).
**Dimension 2: Wood burning**	
5. Wood heating during cold season	Using a heating system (main, fill, background or emergency) using wood at-home (yes, no).
6. Daily use of wood heating (among users)	Number of days per week using a wood heating system during the cold season (7 *vs.* <7).
7. Age of wood-heating device (≥25 years)	Age of the wood heating system (<10, 11–24, ≥25).
8. Lack of knowledge of EPA certification (among users)	Already heard about EPA certification of wood heating system (yes, no).
9. Bonfire at-home during summer	Completion of ≥ one external fire (e.g., campfire, outdoor fireplace) at-home during the last year (yes, no).
**Dimension 3: Heat stress**	
10. Living in a heat island (Sherbrooke only)	Household located with six-digit postal code during interview and coupled with 2011 Canadian census data.
11. No air conditioning	Using air-conditioning system during the hot season (yes, no).
**Dimension 4: ETS**	
12. ETS exposure	Presence of ≥ one person smoking inside the home almost every day (yes, no).
13. ETS exposure among family with children aged 0 to 5	Presence of ≥ one person smoking inside the home almost every day among families with young children aged 0 to 5 (yes, no).
14. ETS exposure among non-smokers	Presence of ≥ one person smoking inside the home almost every day among non-smokers (yes, no).
**Dimension 5: Community noise**	
15. Community noise annoyance	Presence of annoying noise from outside home (often or always *vs.* rarely or never).
16. Annoyance from road-traffic noise	Presence of annoying noise from road-traffic home (often or always *vs.* rarely or never).
17. High density of high-traffic roadways (above median)	Household located using the six-digit postal code during interview and coupled with 2011 Canadian census data.
18. Noise-related concentration disturbances	Presence of annoying noise from outside home who affect the ability to concentrate on daily activities (yes, no).
19. Noise-related sleep problems	Presence of annoying noise from outside home who affect the ability to sleep (yes, no).

Notes: CM = Carbon Monoxide; EPA = Environmental Protection Agency; ETS = Environmental Tobacco Smoke.

#### 2.4.2. Indicators of Social Position

The social position of participants was captured with two socioeconomic indicators and two geographic indicators: 

*Socioeconomic indicators.* The annual household income (˂$20,000; $20,000–49,999; $50,000-79,999; ≥$80,000) and the highest completed education (primary, secondary, college, university) were self-reported. Adults aged between 18 to 24 years old (*n* = 121, 6.1% of the sample) were excluded from analyses examining associations between education and housing-related issues due to possible non-completed schooling.

*Geographic indicators.* Two indicators of social position were computed using participants’ six-digit postal code coupled with 2011 Canadian census data. A composite measure of material deprivation (quintiles) and of population density (people by square kilometers; p/km^2^) for each local community (quartiles; quartile 1 = 3.0–18.2 p/km^2^, quartile 2 = 21.9–255.8 p/km^2^, quartile 3 = 261.2–1,278.2 p/km^2^, and quartile 4 = 1,294.6–5,668.3 p/km^2^) were generated. Population density was used as a proxy for the rural-urban continuum. The composite measure of material deprivation was part of the *Quebec material and social deprivation index* which stems from a principal component analysis done across the province of Quebec [53]. This measure was computed from three proportions specific to the area under investigation: (1) the proportion of individuals 15 years of age and older without a high school diploma, (2) the mean income of individuals 15 years of age and older and (3) the unemployment rate within this age cohort [53].

### 2.5. Statistical Analyses

We first assessed the prevalence of each housing-related issue. We then examined these prevalences as a function of socioeconomic (*i.e.*, annual household income, education level) and geographic (*i.e.*, local community population density, material deprivation) indicators of social position. Summary measures of inequalities (*i.e.*, prevalence ratio and difference with 95% confidence interval) and chi-square analyses were calculated to assess differences across social groups. Significant differences are highlighted in bold print in the tables. Analyses were performed using SPSS software and were weighted for age, sex, education, and household size where appropriate.

## 3. Results

### 3.1. Participant and Regional Characteristics

As mentioned above, half of the participants lived in Sherbrooke (*n* = 1,000) with the remaining living elsewhere in the region (*n* = 1,000). Within each of the 66 Eastern Townships local communities, there were between 11 and 79 participants (mean number of 30.2 participants per community). Participants’ sociodemographic characteristics were different whether they lived in Sherbrooke or outside this town (results not shown). Participants living outside Sherbrooke had increased odds of speaking English as first language (6.3% *vs.* 2.1%, χ^2^ < 0.001), and to be born in Canada (97.0% *vs.* 94.6%, χ^2^ = 0.008). They were also more likely to be less-educated (64.7 % do not have more than completed high school *vs.* 46.3%, χ^2^ < 0.001), and to had a lower household income (56.1% had an income ˂$50,000 *vs.* 52.1, χ^2^ < 0.003).

The housing-related issues of dimension #1 (carbon monoxide), #2 (wood heating), and #3 (heat stress) were the most prevalent in Eastern Townships (results not shown). More than half of households reported at least one source of CM at-home even though half of these did not have any CM detector to monitor potential leaks. Wood heating during winter, by far the most frequently reported source of CM at-home, was reported in one of three households in Eastern Townships. Among these households, half reported using this type of heating system daily and the majority had never heard about the Environmental Protection Agency (EPA) certification for these types of heating systems. More than 60% of households did not have an air-conditioning system. Other frequent housing-related issues characterizing the Eastern Township residents were ETS exposure (23%) and sleep-related problems due to community noise (22%).

### 3.2. Social Inequalities in Housing-related Issues

Prevalence of 13 housing-related issues out of 19 was significantly higher among low-income households (<$20,000) as compared to more affluent ones (≥$80,000; Table 2). Only few issues were more frequent among better-off households, including having at least one source of CM at-home, using a wood heating system during the cold season, and doing bonfires at-home during summer. However, even though affluent households were more likely to use a wood heating system during winter, they were also more likely than their less-advantaged counterparts to use it occasionally (*vs.* daily), to be aware of EPA certifications and to possess a CM detector at-home. The magnitude of social inequalities related to annual household income varied strongly according to housing-related issues. The highest prevalence ratio observed between least and most affluent households was for ETS exposure among families with young children (PR = 4.46). Other prevalence ratios suggesting strong inequalities (PR > 2.50) were noted for noise-related concentration disturbances, ETS exposure (overall), and living in an urban heath island.

**Table 2:** Prevalence of housing-related issues according to annual household income, Eastern Townships, Québec (Canada), 2011 (weighted data).

Housing-related Issues	Annual Household Income				x^2^	PR (95% CI)	PD
	<$20,000	$20,000–49,999	$50,000–79,999	≥$80,000			
	P1 (%)	P2 (%)	P3 (%)	P4 (%)	*p*	P1/P4	P1–P4
**Dimension 1: CM**							
1. CM source(s)	**40.6**	**52.7**	**63.6**	**77.9**	**0.001**	**0.52** **(0.41–0.66)**	**−37.3**
2. Lack of knowledge of CM source(s)	**81.6**	**72.9**	**57.3**	**55.5**	**0.001**	**1.47** **(1.24–1.80)**	**26.1**
3. No CM detector	**78.4**	**66.9**	**53.2**	**46.8**	**0.001**	**1.68** **(1.35–2.09)**	**31.6**
4. No CM detector among houses with CM source(s)	**64.4**	**49.9**	**37.8**	**37.8**	**0.001**	**1.70** **(1.29–2.25)**	**26.6**
**Dimension 2: Wood burning**							
5. Wood heating during cold season	**20.4**	**32.7**	**40.0**	**46.4**	**0.001**	**0.44** **(0.30–0.68)**	**−26.0**
6. Daily use of wood heating (among users)	**59.8**	**48.7**	**51.4**	**35.7**	**0.009**	**1.68** **(1.25–2.25)**	**24.1**
7. Age of wood-heating device (≥25 years)	16.4	16.1	16.5	15.4	0.843	1.06 (0.52–2.02)	1.0
8. Lack of knowledge of EPA certification (among users)	**83.6**	**72.0**	**60.9**	**55.4**	**0.003**	**1.51** **(1.26–1.84)**	**28.2**
9. Bonfire at-home during summer	**14.2**	**23.7**	**39.1**	**42.7**	**0.001**	**0.33** **(0.21–0.54)**	**−28.5**
**Dimension 3: Heat stress**							
10. Living in a heat island (in Sherbrooke only)	**17.4**	9.2 *	5.6 *	4.8 *	**0.001**	**3.62** **(1.49–8.43)**	**12.6**
11. No air conditioning	**66.0**	**61.3**	**58.0**	**56.9**	**0.029**	**1.16** **(0.94–1.46)**	**9.1**
**Dimension 4: ETS**							
12. ETS exposure	**30.8**	26.0 *	**18.8**	10.7 *	**0.001**	**2.88** **(1.59–5.20)**	**20.1**
13. ETS exposure among family with children aged 0 to 5	48.2 *	31.6 **	3.5 **	10.8 **	**0.001**	**4.46** **(2.72–7.47)**	**37.4**
14. ETS exposure among non-smokers	12.5 *	13.8 *	12.7 *	7.0 *	0.552	1.79 (0.75–4.25)	5.5
**Dimension 5: Community noise**							
15. Community noise annoyance	**15.9**	17.4 *	**13.6**	8.6 *	**0.002**	**1.85** **(0.86–3.97)**	**7.3**
16. Annoyance from road-traffic noise	**43.3**	48.2 *	**44.2**	39.6 *	**0.010**	**1.09** **(0.79–1.52)**	**3.7**
17. High density of high-traffic roadways (above median)	55.2	46.3	53.3	47.0	0.096	1.17 (0.89–1.52)	8.2
18. Noise-related concentration disturbances	**16.8**	10.5 *	**9.6**	6.5 *	**0.001**	**2.58** **(1.14–5.87)**	**10.3**
19. Noise-related sleep problems	**23.4**	**22.3**	**25.0**	**14.8**	**0.033**	**1.58** **(0.88–2.83)**	**8.6**

Notes: * = Coefficient of variation between 16.6 and 33.3; ** = Coefficient of variation > 33.3; 95% CI = 95 % Confidence Interval; CM = Carbon Monoxide; EPA = Environmental Protection Agency; ETS = Environmental Tobacco Smoke; P1 to P4 = Proportion #1 to Proportion #4; PD = Prevalence Difference; PR = Prevalence Ratio.

There were nine housing-related issues for which we observed a significant difference according to the education level of the participants (Table 3). Six times out of nine, we observed positive educational gradients, or decreasing prevalence as education increased. This was the case for a lack of knowledge about at-home CM sources and about EPA certification as well, for daily use of wood heating systems, and for the three issues related to ETS exposures where the measured prevalence ratios were the strongest (ranging from 4 to 11). Conversely, inverse educational gradients (*i.e.*, higher prevalence among most educated individuals) were observed for: (1) having bonfires at-home during summer, (2) noise-related sleep problems, and (3) living in a local community above the median for density of high-traffic roadways.

**Table 3:** Prevalence of housing-related issues according to education among participants aged at least 25 years old, Eastern Townships, Québec (Canada), 2011 (weighted data).

Housing-related Issues	Highest Completed Academic Degree				x^2^	PR (95% CI)	PD
	Primary	Secondary	College	University			
	P1 (%)	P2 (%)	P3 (%)	P4 (%)	*p*	P1/P4	P1–P4
**Dimension 1: CM**							
1. CM source (s)	57.1	54.1	53.2	59.9	0.167	0.95 (0.76–1.22)	−2.8
2. Lack of knowledge of CM source (s)	**72.1**	**66.1**	**62.8**	**63.2**	**0.016**	**1.14** **(0.94–1.38)**	**8.9**
3. No CM detector	63.0	63.6	64.8	59.4	0.342	1.06 (0.85–1.32)	3.6
4. No CM detector among houses with CM source (s)	46.7	43.1	45.1	45.8	0.792	1.02 (0.76–1.39)	0.9
**Dimension 2: Wood burning**							
5. Wood heating during cold season	35.2	33.2	32.6	30.8	0.501	1.14 (0.77–1.70)	4.4
6. Daily use of wood heating (among users)	**50.8**	**56.3**	**43.6**	**34.8**	**0.001**	**1.46** **(1.02–2.02)**	**16.0**
7. Age of wood-heating device (≥25 years)	18.8 *	14.6	15.4 *	21.1	0.238	0.89 (0.51–1.55)	−2.3
8. Lack of knowledge of EPA certification (among users)	**74.9**	**72.9**	**57.4**	**58.8**	**0.001**	**1.27** **(1.05–1.55)**	**16.1**
9. Bonfire at-home during summer	**18.8**	**30.2**	**32.1**	**26.6**	**0.001**	**0.71** **(0.42–1.18)**	**−7.8**
**Dimension 3: Heat stress**							
10. Living in a heat island (in Sherbrooke only)	9.1 *	10.2	8.9 *	9.3	0.993	0.98 (0.41–2.34)	−0.2
11. No air conditioning	62.2	58.5	64.7	62.9	0.564	0.99 (0.80–1.23)	−0.7
**Dimension 4: ETS**							
12. ETS exposure	**29.5**	**27.3**	**17.0**	**7.4**	**0.001**	**3.99** **(2.03–7.81)**	**22.1**
13. ETS exposure among family with children aged 0 to 5	54.3 *	18.2 **	9.8 **	5.1 **	**0.001**	**10.6** **(5.79–10.65)**	**49.2**
14. ETS exposure among non-smokers	13.8 *	15.6 *	8.9 *	3.2 *	**0.001**	**4.31** **(1.49–12.52)**	**10.6**
**Dimension 5: Community noise**							
15. Community noise annoyance	14.4	14.9	12.0	13.9	0.562	1.04 (0.52–2.05)	0.5
16. Annoyance from road-traffic noise	41.6	46.3	44.2	43.4	0.058	0.96 (0.69–1.31)	−1.8
17. High density of high-traffic roadways (above median)	**47.3**	**47.9**	**51.2**	**55.6**	**0.017**	**0.85** **(0.66–1.13)**	**−8.3**
18. Noise-related concentration disturbances	8.5 *	11.6	10.6	12.3	0.342	0.69 (0.30–1.57)	−3.8
19. Noise-related sleep problems	**16.4**	**22.5**	**27.0**	**21.5**	**0.008**	**0.76** **(0.43–1.36)**	**−5.1**

Notes: * = Coefficient of variation between 16.6 and 33.3; ** = Coefficient of variation > 33.3; 95% CI = 95 % Confidence Interval; CM = Carbon Monoxide; EPA = Environmental Protection Agency; ETS = Environmental Tobacco Smoke; P1 to P4 = Proportion #1 to Proportion #4; PD = Prevalence Difference; PR = Prevalence Ratio.

### 3.3. Geographic Inequalities in Housing-related Issues

There was a significant gradient as a function of local community material deprivation for eight examined issues (Table 4). Six times out of eight, these gradients were positive, and showed higher prevalence for the most deprived communities (quintile 5). This was the case for using wood fired heating systems during winter (and its daily use), having bonfire at-home during summer, having no-air-conditioning systems, and being exposed to ETS (overall and among families with young children). Inverse geographic gradients (*i.e.*, higher prevalence among least deprived communities, quintile 1) were observed for living (1) in urban heat islands, and (2) among local communities above the median for density of high-traffic roadways.

**Table 4:** Prevalence of housing-related issues according to local community material deprivation, Eastern Townships, Québec (Canada), 2011 (weighted data).

Housing-related Issues	Quintile of Local Community Material Deprivation					x^2^	PR (95% CI)	PD
	Q1 (Least Deprived)	Q2	Q3	Q4	Q5 (Most Deprived)			
	P1 (%)	P2 (%)	P3 (%)	P4 (%)	P5 (%)	*p*	P5/P1	P5–P1
**Dimension 1: CM**								
1. CM source (s)	53.9	53.2	53.9	57.3	60.6	0.303	1.12 (0.85–1.43)	6.7
2. Lack of knowledge of CM source (s)	63.0	66.2	68.1	66.0	68.9	0.149	1.09 (0.90–1.33)	5.9
3. No CM detector	60.7	62.7	62.5	64.1	63.5	0.832	1.05 (0.84–1.30)	2.8
4. No CM detector among houses with CM source (s)	39.7	42.1	44.3	50.1	50.6	0.122	1.27 (0.94–1.73)	10.9
**Dimension 2: Wood burning**								
5. Wood heating during cold season	**24.9**	**27.4**	**31.2**	**36.4**	**43.9**	**0.001**	**1.76** **(1.19–2.61)**	**19.0**
6. Daily use of wood heating (among users)	29.4 *	**40.6**	**36.7**	**60.5**	**62.9**	**0.001**	**2.14** **(1.56–2.92)**	**33.5**
7. Age of wood-heating device (≥25 years)	15.5 *	15.2 *	18.5 *	14.5 *	18.5	0.829	1.19 (0.65–2.21)	3.0
8. Lack of knowledge of EPA certification (among users)	60.8	60.8	70.5	72.8	70.7	0.062	1.16 (0.95–1.42)	9.9
9. Bonfire at-home during summer	**19.2**	**23.2**	**27.7**	**28.0**	**33.2**	**0.001**	**1.73** **(1.07–2.79)**	**14**
**Dimension 3: Heat stress**								
10. Living in a heat island (in Sherbrooke only)	6.2 *	**12.4**	**12.6**	**10.4**	5.9 *	**0.001**	**0.95** **(0.32–2.84)**	**−0.3**
11. No air conditioning	**57.8**	**59.3**	**57.2**	**63.6**	**67.1**	**0.005**	**1.16** **(1.04–1.40)**	**9.3**
**Dimension 4: ETS**								
12. ETS exposure	**13.7**	**17.6**	**25.8**	**25.9**	**28.1**	**0.001**	**2.05** **(1.17–3.60)**	**14.4**
13. ETS exposure among family with children aged 0 to 5	5.0 **	17.2 **	20.2 **	23.3 *	27.3 *	**0.037**	**5.46** **(2.51–11.87)**	**22.3**
14. ETS exposure among non-smokers	7.6 *	8.2 *	11.8 *	12.3 *	16.7	0.052	2.19 (1.00–4.81)	9.1
**Dimension 5: Community noise**								
15. Community noise annoyance	12.2	11.2	13.9	14.6	18.1	0.131	1.48 (0.76–2.88)	5.9
16. Annoyance from road-traffic noise	39.4	39.5	49.1	44.3	47.4	0.167	1.20 (0.88–1.65)	8.0
17. High density of high-traffic roadways (above median)	**56.4**	**75.3**	**53.9**	**51.6**	**14.1**	**0.001**	**0.25 ** **(0.16–0.39)**	**−42.3**
18. Noise-related concentration disturbances	10.5	9.7	10.8	12.3	11.2	0.751	1.07 (0.48–2.36)	0.7
19. Noise-related sleep problems	19.2	22.5	18.8	25.2	21.6	0.498	1.13 (0.65–1.95)	2.4

Notes: * = Coefficient of variation between 16.6 and 33.3; ** = Coefficient of variation > 33.3; 95% CI = 95 % Confidence Interval; CM = Carbon Monoxide; EPA = Environmental Protection Agency; ETS = Environmental Tobacco Smoke; P1 to P5 = Proportion #1 to Proportion #5; PD = Prevalence Difference; PR = Prevalence Ratio; Q1 to Q5 = Quintile #1 to Quintile #5.

Finally, there were 16 housing-related issues for which we observed a significant geographic gradient as a function of local community population density (Table 5). Households located in most densely populated areas (quartile 4) were more frequently affected by community noise issues, urban heat islands, lack of knowledge about CM sources, absence of CM detector at-home (overall and among houses with CM sources), and by old wood fired heating systems. Conversely, households located in the least densely populated areas (quartile 1) had increased odds of having at least one source of CM at-home, being exposed to ETS (among families with young children), and having no air-conditioning systems. These households were also much more prone to having wood fired heating systems (PR = 7.14 or 1/0.14), to use this type of heating system on a daily basis (PR = 5.26 or 1/0.19), and to have bonfire at-home during summer (PR = 5.88 or 1/0.17). 

**Table 5:** Prevalence of housing-related issues according to local community population density, Eastern Townships, Québec (Canada), 2011 (weighted data).

Housing-related Issues	Quartile of Local Community Population Density (Persons/km^2^)				x^2^	PR (95% CI)	PD
	Q1 (3.0–18.2)	Q2 (21.9–255.8)	Q3 (261.2–1,278.2)	Q4 (1,294.6–5,668.3)			
	P1 (%)	P2 (%)	P3 (%)	P4 (%)	*p*	P4/P1	P4–P1
**Dimension 1: CM**							
1. CM source (s)	**73.0**	**66.6**	**46.1**	**38.6**	**0.001**	**0.53** **(0.41–0.68)**	**−34.4**
2. Lack of knowledge of CM source (s)	**64.9**	**63.8**	**64.8**	**77.5**	**0.001**	**1.19** **(1.00–1.43)**	**12.6**
3. No CM detector	**56.4**	**55.0**	**63.6**	**75.8**	**0.008**	**1.34** **(1.10–1.64)**	**19.4**
4. No CM detector among houses with CM source (s)	**46.6**	**41.2**	**41.7**	**56.4**	**0.001**	**1.21** **(0.92–1.59)**	**9.8**
**Dimension 2: Wood burning**							
5. Wood heating during cold season	**57.4**	**43.6**	**24.1**	**7.9**	**0.001**	**0.14** **(0.08–0.23)**	**−48.5**
6. Daily use of wood heating (among users)	**62.7**	**45.1**	**32.5**	11.9 **	**0.001**	**0.19** **(0.12–0.29)**	**−50.8**
7. Age of wood-heating device (≥ 25 years)	**17.2**	12.7 *	18.5 *	26.0 *	**0.003**	**1.51** **(0.88–2.58)**	**8.8**
8. Lack of knowledge of EPA certification (among users)	72.9	65.0	59.2	74.7	0.139	1.02 (0.87–1.21)	1.8
9. Bonfire at-home during summer	**43.4**	**32.9**	**22.8**	**7.3**	**0.001**	**0.17** **(0.09–0.31)**	**−36.1**
**Dimension 3: Heat stress**							
10. Living in a heat island (in Sherbrooke only)	**0.0**	**0.0**	**7.1**	**30.1**	**0.001**	**-**	**30.1**
11. No air conditioning	**69.5**	**63.1**	**57.3**	**54.5**	**0.001**	**0.78** **(0.63–0.98)**	**−15.0**
**Dimension 4: ETS**							
12. ETS exposure	25.9	18.6	20.7	24.5	0.091	0.95 (0.59–1.53)	−1.4
13. ETS exposure among family with children aged 0 to 5	30.1 *	14.8 **	11.5 **	13.8 **	**0.028**	**0.46** **(0.26–0.79)**	**−16.3**
14. ETS exposure among non-smokers	15.4	9.0 *	9.7 *	10.7 *	0.481	0.69 (0.34–1.43)	−4.7
**Dimension 5: Community noise**							
15. Community noise annoyance	**11.5**	**9.2**	**16.7**	**19.1**	**0.001**	**1.66** **(0.85–3.23)**	**7.6**
16. Annoyance from road-traffic noise	**40.8**	**39.5**	**48.7**	**47.9**	**0.002**	**1.17** **(0.86–1.60)**	**7.1**
17. High density of high-traffic roadways (above median)	**0.0**	**48.6**	**80.7**	**75.9**	**0.001**	**-**	**75.9**
18. Noise-related concentration disturbances	**8.2**	**9.6**	**11.8**	**14.4**	**0.003**	**1.76** **(0.79–3.90)**	**6.2**
19. Noise-related sleep problems	**17.2**	**19.3**	**22.7**	**27.8**	**0.001**	**1.62** **(0.96–2.73)**	**10.6**

Notes: * = Coefficient of variation between 16.6 and 33.3; ** = Coefficient of variation > 33.3; 95% CI = 95 % Confidence Interval; CM = Carbon Monoxide; EPA = Environmental Protection Agency; ETS = Environmental Tobacco Smoke; P1 to P4 = Proportion #1 to Proportion #4; PD = Prevalence Difference; PR = Prevalence Ratio; Q1 to Q4 = Quartile #1 to Quartile #4.

## 4. Discussion

This study aimed to examine the socioeconomic and geographic gradients in the distribution of various housing-related issues on regional and local scales. Overall, nearly all housing-related issues were more frequent among low socioeconomic status households compared to more advantaged ones, with some prevalence ratios suggesting unexpectedly high levels of inequality including ETS exposure among families with young children for which the prevalence ratio (low *versus* high education) exceeded 10. Similar findings were noted when using geographic indicators of social position. A higher prevalence of housing-related issues was commonly observed among most materially deprived or densely populated local communities in the Eastern Townships.

### 4.1. Social Inequalities in Housing-related Issues

On a regional level, income appears to be a major determinant of better housing and residential conditions, and ultimately health and well-being. As previously observed by other authors, our results suggest lower proportions of air-conditioning systems among less affluent households [54] as well as exposure to higher levels of noise [4,27], road-traffic [27], air pollutant [54], and ETS exposure [3,4]. Our findings were then supported by scientific literature on residential segregation, which highlights that economically deprived households tend to cluster in neighborhoods where housing prices are cheaper [4,7,24,25,26,55]. In the light of these results, it appears that regional and local public health interventions targeting housing-related issues may be more beneficial if they were spread in neighborhoods where housing prices are lower.

Along with disposable income, level of education was also found to be an important determinant for better housing and residential conditions. In this study, education seemed to capture a different construct than income, given that many people with university degrees had low or moderate income (and *vice versa*). Unsurprisingly, more educated adults had a better knowledge about at-home CM sources and EPA certification of wood fired heating systems. These adults were also less likely to be exposed to ETS. These results are in line with prior studies suggesting a higher exposition to ETS among less educated people [4,56]. Similar to our study, the prevalence ratio between less *vs.* more educated parents of ETS exposure at-home was found to be as high as 11 in one recent Danish study [57]. These impressively high magnitudes suggest that social inequalities related to environmental health issues (e.g., ETS exposure) may be even greater than for individual health behavior (e.g., smoking). These results also highlight the importance of facilitating access to education among more vulnerable groups as a way to reduce inequalities in health.

### 4.2. Geographic Inequalities in Housing-related Issues

Disparities were also observed in housing-related issues as a function of geographic indicators of social position (*i.e.*, material deprivation and residential density). It is worth noting that these two indicators were not strongly correlated (*r* = −0.06). The use of wood fired heating systems (overall and daily), the realization of bonfires at-home during summer, having no air-conditioning systems at-home, and ETS exposure (overall and among families with young children) were all more prevalent among the most deprived local communities. Because the material deprivation index of local communities is based on participants’ education and income [53], higher ETS exposure, and frequent lack of air-conditioning systems at-home among most deprived communities were congruent with patterns of social inequalities (Table 2 and Table 3). This suggests that these gradients may be attributable to individual differences rather than contextual ones. Higher proportions of wood fired heating systems and bonfires at-home during summer in the most deprived local communities may however be specific to contextual characteristics. Bonfire is indeed usually prohibited for tenants of building rentals (which are far more frequent in central urban neighborhoods). Furthermore, wood fired heating systems are usually less present within this type of building. This may contribute to negative gradients between most and least deprived local communities. Public health interventions which tackle the negative outcomes associated with these issues will have to take account of this contextual effect.

We finally observed inequalities according to population density. Issues related to community noise were unsurprisingly increased in more densely populated areas. Another issue characterizing such communities was living in urban heat islands. These latter results support that population density is an appropriate proxy for the rural-urban continuum, as noise and heat islands are recognized as being more frequent in urban areas, especially in urban cores. We also found that fewer urban participants had a CM detector at home, which is of concern given the risk of residential CM poisoning associated with blasting operations in construction projects. Housing-related issues with higher prevalence among less densely populated areas were related to wood heating systems, bonfires at-home, absence of air-conditioning systems, and the presence of CM sources. It was observed in literature that wood burning is more common among rural settlements, which are usually less densely populated [7]. It is thus not surprising to note that the presence of CM sources at-home were also more important in such areas (wood fired heating systems are the most frequent CM source documented in our region). To our knowledge, there is no literature on the associations between air-conditioning systems and population density. There is however one report that indicates higher proportions of such systems among more affluent urban households [25]. We then proposed two hypotheses to explain this lower proportion of air-conditioning systems among less densely populated areas. Maybe such systems are less relevant due to scarcity of heat islands in less densely populated areas. This may also be related to the lower income of such households as proposed by one study [54].

### 4.3. Strengths and Limitations

Based on the WHO *Commission on the Social Determinants of Health* framework, this study significantly contributes to the field of surveillance in environmental health inequalities. This work however contains limitations. A selection bias may have arisen from the exclusion of people with mobile phone only, and of other without any phone at all [45]. These proportions were respectively estimated to 13.0% and 1.1% in 2010 by Statistics Canada [58]. It was also found that these people are generally more likely to be younger males living in small households, and having a low income, as compared to the Canadian population [59]. ETS exposure may also be underestimated due to social desirability [60]. While heads of households’ (*i.e.*, the person who is the most familiar with household concerns) are usually selected for telephone surveys on housing conditions [27], we used randomly selected participants. This may have introduced information bias for specific issues such as those related to the characteristics of wood heating systems. Finally, our data regarding community noise was self-reported and it is increasingly recommended to use objective data to reduce error measurements [31]. However, we think that perceived noise is at least as important that objective noise when studying the health impact of housing and residential conditions [61]. 

## 5. Conclusions

We concluded that there are several inequalities in housing and/or residential issues on regional and even local scales. Not only such inequalities exist but they were much deeper than we expected. We think these results raise the need to: (1) increase efforts to monitor such inequalities, (2) advocate for awareness and political will among municipal officials, and (3) foster partnerships between environmental health and health promotion in order to build appropriate strategies to struggle such inequalities. In addition to individual behaviors and access to care, this study highlights that physical environments, and housing conditions in particular, may play an important intermediate role between social position and health. Unhealthy housing conditions are distributed as a function of various socioeconomic and geographic factors and this contribute to widen health inequalities. Public health actors should take account of these results and increase their efforts to struggle inequalities in housing and residential conditions. Not only actions that target physical environments may have a larger impact on population health, they may also protect people involuntarily exposed to hazards (e.g., children exposed to ETS). Towards this task, regional surveillance is essential to mobilize local stakeholders and target vulnerable groups. Finally, and perhaps the most important thing to remember, any examined housing-related issue alone is more or less relevant for the understanding of health inequalities and/or the building of specific interventions. However, all together, they suggest that different social positions may positively or negatively impact health throughout various exposures to different housing-related issues.
